# Synovial fibroblasts display an uncontrolled inflammatory and tissue destructive response to TNF-α

**DOI:** 10.1186/ar3591

**Published:** 2012-02-09

**Authors:** George D Kalliolias, Janice Chen, Galina Grigoriev, Lionel B Ivashkiv

**Affiliations:** 1 Research Department, Arthritis and Tissue Degeneration Program, Hospital for Special Surgery, New York, NY, 10021, USA; 2 Medicine, Weill Cornell Medical College, New York, NY, 10021, USA; 3 Graduate Program in Immunology and Microbial Pathogenesis, Weill Cornell Graduate School of Medical Sciences, New York, NY, 10021, USA

## Background

Synovial fibroblasts are key players in the pathogenesis of Rheumatoid Arthritis (RA) and potentially attractive treatment targets. Upon activation within the joint's inflammatory milieu, they gain a transformed phenotype and produce pro-inflammatory cytokines (mainly IL-6) and tissue destructive enzymes [1].

## Materials and methods

Synovial fibroblasts were isolated via enzymatic processing from synovial tissues obtained from patients with RA or Osteoarthritis (OA). Synovial fibroblasts (passages 2-4) were stimulated with TNF-α (10 ng/ml) only on day 1. The expression of TNF-α-target genes was measured by qPCR in time course experiments (1, 3, 6, 24, 48, 72, 96 and 120 hours after TNF-α stimulation).

Human macrophages (Mϕ) generated in vitro (blood derived CD14+ cells stimulated for 48 h with M-CSF) were used in similar time course experiments as controls.

## Results

In Mϕ it was observed a rapid (within 1-3 hours) induction of TNF-α-target genes (including *TNF-a*, *IL-1β*, *IL-6 *and *IL-8*) that was restrained back to the baseline within a few hours (3-24 hours depending on the gene). In stark contrast, synovial fibroblasts displayed a remarkably more sustained response to TNF-α. *IL-6 *mRNA expression was induced within a few hours by TNF-α, and induction increased continuously for 72-96 h despite the absence of any further exogenous TNF-α stimulation. The levels of *IL-6 *mRNA induced by TNF-α in synovial fibroblasts were substantially higher compared to human Mϕ, suggesting that within the joint microenvironment, synovial fibroblasts and not Mϕ are the main source of IL-6. By adding the supernatants from 96 h TNF-α-stimulated fibroblast cultures on unstimulated synovial fibroblasts, a similar robust induction of *IL-6 mRNA *was observed, suggesting that there is a TNF-α-induced soluble factor that mediates the sustained response. A similar pattern of sustained expression was observed for other TNF-α-target genes including *IL-1β*, *IL-8 *and *MMPs*. Interestingly, there was no difference between OA- and RA-derived synovial fibroblasts in their response to TNF-α.

## Conclusions

In contrast to human Mϕ, synovial fibroblasts display a sustained inflammatory and tissue destructive response to TNF-α. Our observations suggest that synovial fibroblasts may lack the homeostatic mechanisms that control and terminate the effects of TNF-α on human Mϕ [2]. To support this hypothesis, further investigation is needed at the level of proximal and distal TNF-α signaling events and at the level of epigenetic regulation of TNF-α-target genes in synovial fibroblasts.
